# P03-013 - Symptomatic neuromuscular sarcoidosis

**DOI:** 10.1186/1546-0096-11-S1-A210

**Published:** 2013-11-08

**Authors:** M Mulazzani, C Haberler, F Zimprich, EV Hartl, H Cetin

**Affiliations:** 1 Department of Neurology, Medical University of Vienna, Vienna, Austria; 2 Institute of Neurology, Medical University of Vienna, Vienna, Austria; 3 Landesklinikum Horn, Horn, Austria

## Introduction

Sarcoidosis is a granulomatous disease possibly affecting all organ systems. Less than 10% of sarcoidosis patients develop symptomatic neurological involvement. Although muscular non-caseating granulomas (NCGs) can be found in up to 75% of sarcoidosis patients, muscular symptoms develop only in less than 0.5% of sarcoidosis patients. Corticosteroids (CS) are the mainstay of medical therapy, but their effect on chronic myopathy is not consistent. Additive immunosuppressive therapy with azathioprine, cyclophosphamide, methotrexate, or chloroquine may be necessary in patients inadequately responding to CSs.

## Case report

A pathological routine x-ray of an asymptomatic 43-year-old farmer led to a pulmonary CT, which showed mediastinal and hilar lymphadenopathy. Sporadic epitheloid cells were found in lymph node biopsy. Bronchoalveolar lavage contained predominantly lymphocytes with a moderately elevated CD4/CD8 ratio (3.6). Tuberculosis-PCR and -culture were negative. Spirometry demonstrated only marginal obstruction. Because of iridocyclitis, prednisolone therapy (100 mg/d for two weeks, 50 mg/d for two months, 25 mg/d for six more months, then 30 mg/d) was initiated. After five months of CS therapy, the patient first experienced stabbing pain (up to 3 out of 10 according to the visual analog scale, VAS) in the left thigh without paresis. Six more months later he presented with pain aggravation (VAS: up to 5) and additional moderate paresis of left hip flexion and -extension. MRI demonstrated a slight T2 (TIRM) hyperintensity in the left vastus lateralis muscle. Electromyography showed spontaneous fibrillation and positive sharp waves with normal motor unit potentials. A muscle biopsy illustrated two processes: an inflammatory myopathy with prominent endomysial and perimysial infiltrations (mainly CD4-lymphocytes, some multinucleated giant cells and epitheloid cells) and pronounced neurogenic changes (grouplike atrophy of muscle fibers and florid disintegration of muscle fibers). Laboratory analysis showed elevated creatine kinase (893 U/l with an upper limit (UL) of 200 U/l), and lysozyme (22.1 mg/l with UL of 17.6). Four weeks after the muscle biopsy, he still suffered from stinging pain in the left vastus lateralis muscle sensitive to activity and pressure (VAS: up to 8) despite medical therapy with diclofenac (150 mg/d) and hydromorphone (4-5 mg/d). He had no signs of arthritis or of cardiac, pulmonary, and visual involvement; lupus pernio was not visible. Oral methylprednisolone was increased to 60 mg per day. Five weeks later, he additionally presented stabbing pain in the left semitendinosus muscle with new pain-related moderate paresis of foot dorsal extension, foot plantar flexion and adduction of the left hip. Another inpatient stay for MRI, EMG, NCS, nerve biopsy, semitendinosus muscle biopsy, laboratory analysis and additive immunosuppressive therapy is planned (results will be presented on the congress).

## Discussion

Chronic symptomatic neuromuscular sarcoidosis is often refractory to medical therapy and potentially leading to severe and permanent disability. Only little data are available regarding optimal therapy of this rare manifestation of systemic sarcoidosis.

## Competing interests

None Declared
